# Lipoprotein(a) and Cardiovascular Outcomes in Patients With Coronary Artery Disease and Different Metabolic Phenotypes

**DOI:** 10.3389/fcvm.2022.870341

**Published:** 2022-05-20

**Authors:** Jing-Lu Jin, Hui-Wen Zhang, Hui-Hui Liu, Cheng-Gang Zhu, Yuan-Lin Guo, Na-Qiong Wu, Rui-Xia Xu, Qian Dong, Jian-Jun Li

**Affiliations:** ^1^Key Laboratory of Cardiovascular Disease, Cardiometabolic Center, National Center for Cardiovascular Diseases, Fu Wai Hospital, Chinese Academy of Medical Sciences, Peking Union Medical College, Beijing, China; ^2^Department of Endocrinology, Genetics, and Metabolism, Beijing Children’s Hospital, National Center for Children’s Health, Capital Medical University, Beijing, China

**Keywords:** CAD, lipoprotein(a), metabolic phenotypes, coronary severity, outcome

## Abstract

**Background:**

The positive relationship between metabolic healthy obesity (MHO) and cardiovascular risk has been under debate in recent years. Previously, strong evidence supported the causal role of increased plasma lipoprotein(a) [Lp(a)] levels in cardiovascular disease (CVD). The current study aimed to investigate the different associations of Lp(a) and cardiovascular events (CVEs) in patients with coronary artery disease (CAD) and different metabolic phenotypes.

**Methods:**

A total of 5,089 patients who were angiography-proven CAD were consecutively included and followed up for CVEs. Obesity was defined as a body mass index (BMI) ≥25 kg/m^2^ according to Asia-specific BMI criteria. Patients were divided into four groups according to metabolic phenotypes, namely metabolically healthy/unhealthy non-obese and metabolically healthy/unhealthy obese [metabolically healthy non-obese (MHN), MHO, metabolically unhealthy non-obese (MUN), and metabolically unhealthy obesity (MUO)]. Comparisons of CAD severity and outcomes were performed among four groups. Cox regression analyses and cubic spline models were used to examine the relationship between Lp(a) and CVEs in patients with different metabolic phenotypes.

**Results:**

During a median of 7.5 years’ follow-up, 540 (10.6%) CVEs occurred. MUN and MUO populations had more severe coronary stenosis than MHN ones, while no significant difference in the Gensini score (GS) was observed between MHN and MHO. Patients with MUN and MUO presented a higher risk of CVEs than patients with MHN (hazard ratio [*HR*]: 1.414, 95% *CI*: 1.024–1.953–1.556 and *HR*: 1.747, 95% *CI*: 1.295–1.363, *p* < 0.05). In subgroup analysis, restricted cubic spline models showed that there was no association between Lp(a) and CVEs in patients in MHN and MHO, while the MUN and MUO groups presented increasing associations between Lp(a) and CVEs and such association was stronger in the MUO group. In Cox regression analysis, Lp(a) >50 mg/dl was associated with a 2.032- and 2.206-fold higher risk of subsequent CVEs in the MUO and MUN subgroups, respectively.

**Conclusion:**

Among patients with angiography-proven stable CAD, Lp(a) had a more significant prognostic value in both MUO and MUN individuals regardless of obesity, suggesting the importance of screening for cardiovascular risk with Lp(a) in metabolically unhealthy patients.

## Introduction

The prevalence of obesity in Chinese adults has increased significantly, which has become a severe public health issue. The excessive accumulation of body fat in obese individuals was correlated with a surge in rates of several cardiometabolic disorders, such as hypertension, impaired glucose metabolism, hypertriglyceridemia, and low levels of high-density lipoprotein cholesterol (HDL-C). However, some obese individuals have only less than one metabolic disorder and have been categorized as “metabolically healthy obesity” (MHO) (1, 2). As previously reported, MHO is characterized by a proinflammatory phenotype of circulating monocyte subsets (3). In fact, several previous studies have investigated the different relationships between MHO and coronary artery disease (CAD) risk and came to different conclusions. According to previous reports, among metabolic phenotypes, such as MHO, metabolically healthy non-obese (MHN), metabolically unhealthy non-obese (MUN), and metabolically unhealthy obesity (MUO), MHO patients had higher carotid intima-media thickness and a higher risk of heart failure than MHN individuals (4, 5). Moreover, the Atherosclerosis Risk in Communities (ARIC) Study and Evaluation of Chest Pain (PROMISE) study reported controversial results regarding the association between MHO and cardiovascular events (CVEs) (5, 6). Besides, no such study was performed in secondary prevention patients with angiography-proven CAD. Hence, in the current study, approximately 5,000 patients who received coronary angiography were documented. Both coronary severity and outcomes were compared among four metabolic phenotypes.

The full spectrum of risk factors for CAD among patients with different metabolic phenotypes has not been fully determined yet. According to the study by Commodore-Mensah et al. high-sensitivity-cTnT (≥6 ng/L) was associated with a higher risk of cardiovascular disease (CVD) across all metabolic phenotypes (6). Lipid parameters were also crucial in determining the presence and progression of atherosclerosis. Lipoprotein(a) [Lp(a)] is an LDL-like particle that induces proatherogenic and prothrombotic effects *via* LDL moiety and plasminogen-like apolipoprotein(a) (7, 8). Unlike other lipid parameters, circulating Lp(a) levels are rarely influenced by dietary and environmental factors (9). Thereby, in the setting of different metabolic phenotypes, the levels of L(a) may well reflect the CVE risk in patients with established CAD. Thus, we conducted a prospective, relatively large cohort study to investigate the predictive value of Lp(a) levels for the severity and prognosis of CAD in a Chinese population with different metabolic phenotypes.

## Materials and Methods

### Study Population

Our study complied with the Declaration of Helsinki and was approved by the hospital’s ethical review board (Fu Wai Hospital and National Center for Cardiovascular Diseases, Beijing, China). Informed written consent was obtained from all patients enrolled in this study.

As described in Supplementary Figure 1, before February 2015, 6,505 patients who were scheduled for coronary angiography due to angina-like chest pain were documented. The detailed inclusion criteria were described in the flowchart. Among these patients, 498 were excluded because they did not have angiography-proven CAD (coronary stenosis <50% of at least one coronary artery). Other patients were excluded for acute coronary syndrome, decompensated heart failure, severe liver and/or renal insufficiency, thyroid dysfunction, systematic inflammatory disease, malignant disease, and excessive drinking. Finally, 5,089 of them were enrolled in the study. Patients were followed up at 6 months intervals *via* telephone or in-person interviews. Trained nurses or physicians who were blinded to the clinical data fulfilled the interview. The primary endpoints (CVEs) were cardiovascular mortality, non-fatal myocardial infarction (MI), and stroke. Non-fatal MI was diagnosed as positive cardiac troponins along with typical chest pain or typical electrocardiogram (ECG) serial changes. Stroke was diagnosed by the presence of typical symptoms and imaging.

Participants were considered metabolically healthy if they met two of the following four criteria: high triglycerides (TGs ≥ 150 mg/dl) or lipid-lowering drugs, increased systolic blood pressure (SBP ≥ 130 mmHg) or diastolic blood pressure (DPB ≥ 85 mmHg) or prescribed anti-hypertensive drugs, high fasting glucose (≥ 100 mg/dl) or medications for diabetes (insulin and oral antidiabetic), and low HDL-C (<40 mg/dl for men and <50 mg/dl for women) (10).

Diabetes mellitus (DM) was diagnosed by fasting plasma glucose (FPG) ≥130 mg/dl or the 2-h plasma glucose of the oral glucose tolerance test ≥200 mg/dl or currently prescribed antidiabetic medications. Hypertension was defined as self-reported hypertension, and currently prescribed antihypertensive drugs. Information on other diseases, family history, and prior therapy of every patient was collected from self-reported medical history.

### Laboratory Analysis

Blood samples were obtained from each patient from the cubital vein after at least 12-h fasting. In an enzymatic assay, concentrations of total cholesterol (TC), TG, and HDL-C were measured using an automatic biochemistry analyzer (Hitachi 7150, Tokyo, Japan). The calculation of low-density lipoprotein cholesterol (LDL-C) was done using the Friedewald equation: LDL-C = TC-(HDL-C + TG/5). Lp(a) was determined by the immunoturbidimetry method [LASAY Lp(a) auto, SHIMA Laboratories Co., Ltd]. An Lp(a) protein validated standard has been used to calibrate the examination, and the coefficient of variation (CV) value of repetitive measurements was below 10%. The concentrations of glucose were measured by the enzymatic hexokinase method. Hemoglobin A1c (HbA1c) was measured using a Tosoh Automated Glycohemoglobin Analyzer (HLC-723G8, Tokyo, Japan).

### Evaluation of Coronary Artery Disease Severity

Angiographic data were evaluated from catheter laboratory records by three experienced interventional cardiologists as previously reported. The Gensini score (GS) was calculated as the stenosis score by multiplying the location score for all diseased segments. The severity score of each coronary lesion was defined according to the narrowing degree of the coronary artery and its importance (11, 12).

### Statistical Analysis

The statistical analyses were performed using SPSS version 21.0 software (SPSS Inc., Chicago, IL, United States) and STATA SE 11.0 software. The values were expressed as the mean ± SD or median (Q1–Q3 quartiles) for the continuous variables and as a number (percentage) for the categorical variables. The Kolmogorov–Smirnov test was used to test the distribution pattern. The differences in clinical characteristics between groups were analyzed using Student’s *t*-test, Mann–Whitney *U*-test, χ^2^-tests, or Fisher’s exact test where appropriate. The event-free survival rates among groups were estimated by the Kaplan–Meier method and compared by the log-rank test. Univariate and multivariate Cox regression analyses were performed to calculate the hazard ratios (*HR*s). In the multivariate Cox regression models, traditional risk factors, such as age, sex, body mass index (BMI), smoking, and family history of CAD. GS, left ventricular ejection fraction, creatinine, LDL-C, and previous use of statin were used as adjustments. The variables in the definition of the metabolic phenotype were not added in the model. A value of *p* < 0.05 was considered statistically significant.

## Results

### Baseline Characteristics

Baseline demographic and laboratory characteristics of the study population according to the metabolic phenotype are shown in Table 1. Overall, MHN, MHO, MUN, and MUO phenotypes accounted for 13.5, 12.3, 25.8, and 48.0% of the total population, respectively. Metabolically unhealthy individuals were older, had a higher proportion of men, and had higher levels of FPG, HbA1c, TC, LDL-C, TG, and GS but lower levels of HDL-C and Lp(a). Men and current smokers were most likely to be MHO while the MUO population had the highest levels of FPG, HbA1c, TC, LDL-C, TG and the lowest level of HDL-C.

**TABLE 1:** Baseline characteristics.

Variables	Total	MHN	MHO	MUN	MUO	*P*
	*n* = 5089	*n* = 705	*n* = 624	*n* = 1315	*n* = 2445	
**Clinical characteristics**						
Age, years	58.0 ± 10.3	59.7 ± 10.0	56.5 ± 10.3	60.2 ± 9.8	56.8 ± 10.4	<0.001
Male, *n* (%)	3691 (72.5)	534 (75.7)	507 (81.3)	846 (64.3)	1804 (73.8)	<0.001
BMI (kg/m^2^)	25.8 ± 3.0	22.6 ± 1.6	27.4 ± 2.1	23.2 ± 1.4	27.8 ± 2.3	<0.001
Hypertension, *n* (%)	3228 (63.4)	218 (30.9)	256 (41.0)	903 (68.7)	1851 (75.7)	<0.001
DM, *n* (%)	1419 (28.0)	76 (10.8)	64 (10.3)	604 (45.9)	1080 (48.3)	<0.001
Family history of CAD, *n* (%)	734 (14.4)	100 (14.2)	92 (14.7)	171 (13.0)	371 (15.2)	0.341
Current smoker, *n* (%)	2782 (54.7)	388 (55.0)	375 (60.1)	671 (51.0)	1348 (55.1)	0.002
Drinking, *n* (%)	1472 (28.9)	210 (29.8)	183 (29.3)	328 (24.9)	751 (30.7)	0.003
**Laboratory findings**						
Glucose (mg/dL)	102.6 ± 30.6	86.4 ± 16.2	90 ± 12.6	106.2 ± 32.4	108.0 ± 34.2	<0.001
HbA1c (%)	6.4 ± 1.1	5.8 ± 0.6	5.9 ± 0.6	6.5 ± 1.2	6.6 ± 1.4	<0.001
Creatinine (μmoL)	76.0 ± 16.7	75.2 ± 16.7	75.6 ± 13.6	75.0 ± 17.6	76.8 ± 17.0	0.544
TC (mg/dL)	160.22 ± 43.16	156.72 ± 37.72	159.06 ± 39.28	159.44 ± 43.94	162.56 ± 45.11	<0.001
HDL-C (mg/dL)	40.44 ± 10.50	49.00 ± 10.89	46.67 ± 10.50	39.28 ± 10.11	38.11 ± 8.94	<0.001
LDL-C (mg/dL)	98.00 ± 38.38	94.11 ± 37.33	97.22 ± 35.00	97.61 ± 38.11	98.56 ± 40.44	<0.001
TG (mg/dL)	135.00	97.06	105.88	147.35	160.59	<0.00
	(99.71–175.83)	(76.76–120.88)	(85.59–127.06)	(104.12–191.47)	(119.12–210.88)	1
Lipoprotein(a) (mg/dL)	15.7 (6.8–36.2)	18.6 (8.4–39.9)	19.3 (7.7–42.7)	15.8 (7.1–34.8)	14.5 (6.3–34.0)	<0.001
LVEF (%)	63.3 ± 8.3	63.3 ± 8.3	63.8 ± 8.6	63.3 ± 9.0	63.4 ± 7.8	0.200
Gensini score	32 (15–47)	28 (12–38)	30 (14–43)	36 (16–49)	34 (16–49)	<0.001
**Medications**						
Baseline Statins, *n* (%)	3060 (60.1)	494 (70.7)	419 (67.1)	846 (64.3)	1301 (53.2)	<0.001
Follow-up Statins, *n* (%)	4896 (95.5)	694 (98.5)	602 (96.4)	1253 (95.3)	2313 (94.6)	<0.001
Baseline Aspirin, *n* (%)	3114 (61.2)	420 (59.6)	365 (58.5)	805 (61.2)	1523 (62.3)	0.280
Baseline ACEIs/ARBs, *n* (%)	1395 (27.4)	185 (26.2)	180 (28.9)	372 (28.3)	658 (26.9)	0.482
Baseline β-blockers, *n* (%)	2692 (52.9)	375 (53.2)	329 (52.7)	714 (54.3)	1274 (52.1)	0.333

*Data were expressed as mean ± SD, median with 25th and 75th percentile or n (%). BMI, body mass index; DM, diabetes mellitus; HbA1c, hemoglobin A1c; TC, total cholesterol; TG, triglyceride; LDL-C, low-density lipoprotein cholesterol; HDL-C, high-density lipoprotein cholesterol; GS, gensini score; ACEIs, ACE inhibitors; ARBs, angiotensin receptor blockers.*

### Metabolic Phenotype, Lipoprotein(a), and Coronary Severity

**FIGURE 1:**
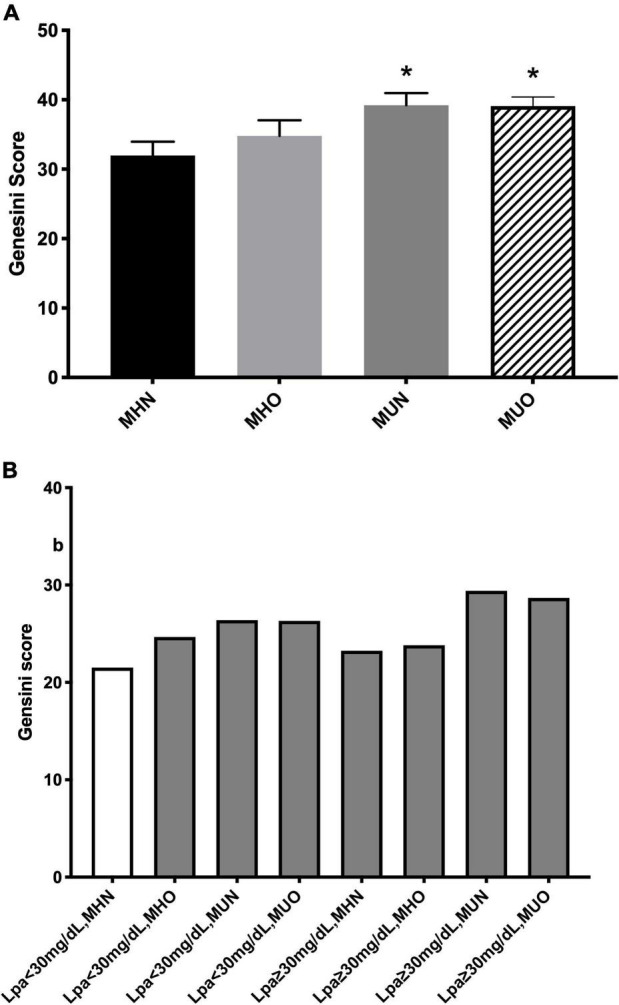
Coronary severity according to **(A)** metabolic phenotype; and **(B)** both status of metabolic phenotype and lipoprotein(a) [Lp(a)]. *p < 0.05.

As shown in Figure 1A, MUN and MUO individuals had more severe coronary stenosis than the MHN ones as assessed using the GS while no significant difference in the GS was observed between the MHN and MHO groups. When Lp(a) was incorporated in the stratification, using Lp(a) <30 mg/dl plus MHN as a reference, Lp(a) <30 mg/dl plus MUN or MUO subgroups had a higher GS, while the most severe coronary stenosis existed in Lp(a) ≥30 mg/dl plus MUN or MUO subgroups (Figure 1B).

### Metabolic Phenotype, Lipoprotein(a), and Cardiovascular Events

Supplementary Table 1 presents the relative risk of CVEs among four metabolic phenotypes. The MUN and MUO subgroups had a 1.414- and 1.747-fold significant higher risk of CVEs (*p* < 0.05) while the MHO subgroup did not present a higher cardiovascular risk (*p* > 0.05).

**FIGURE 2:**
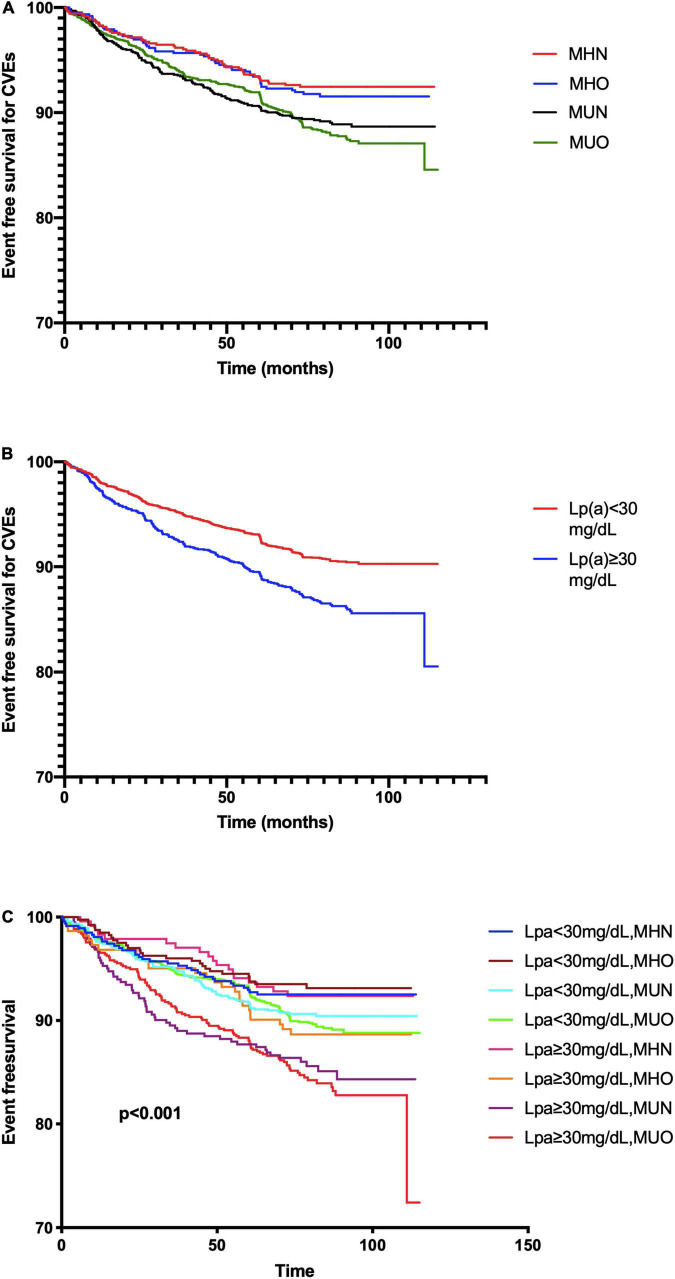
The Kaplan–Meier analysis according to **(A)** metabolic phenotype; **(B)** Lp(a) levels; and **(C)** both status of metabolic phenotype and Lp(a).

The Kaplan–Meier analysis (Figure 2A) showed that MUO and MUN subjects had significantly lower event-free survival rates than MHN ones (*p* < 0.05), while MHO and MHN subgroups presented similar CVE risk. Meanwhile, patients with Lp(a) ≥30 mg/dl showed a higher event rate (Figure 2B). In terms of both metabolic phenotypes and Lp(a) levels, the Lp(a) ≥ 30 mg/dl plus MUN or MUO groups had higher event rates (Figure 2C).

**FIGURE 3:**
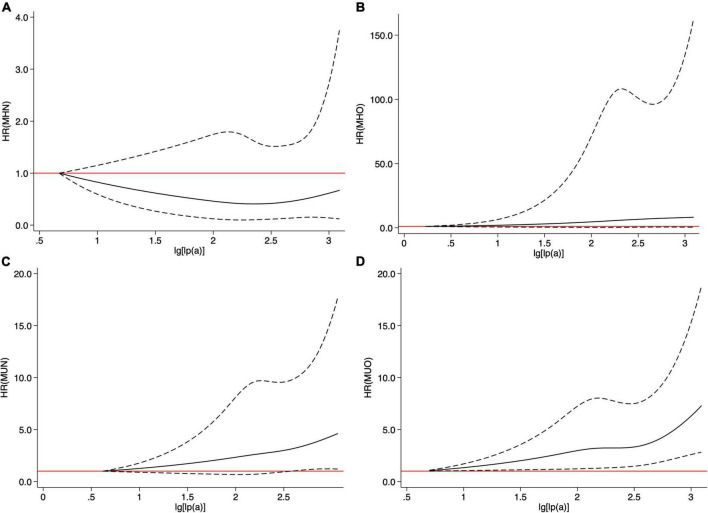
Cubic spline models of Lp(a) in four metabolic phenotypes **(A)** metabolically healthy non-obese (MHN), **(B)** metabolically healthy obesity (MHO), **(C)** metabolically unhealthy non-obese (MUN), and **(D)** metabolically unhealthy obesity (MUO).

Restricted cubic spline models showed that no association between Lp(a) and CVEs existed in the MHN and MHO groups (Figures 3A,B). The MUO and MUN groups had increasing associations between Lp(a) and CVEs, while such association was relatively stronger in the MUO group (Figures 3C,D). Table 2 shows that continuous Lp(a) (per SD increment) was positively associated with CVEs in patients belonging to the MUO and MUN groups (adjusted *HR* per 1-SD increment: 1.236, 95% *CI*: 1.063–1.437 and adjusted *HR* per 1-SD increment: 1.275, 95% *CI*: 1.151–1.412, respectively, all *p* < 0.05) but not in those belonging to the MHO and MHN groups (*p* > 0.05). When Lp(a) was analyzed using more precise cut-off values as 10, 30, and 50 mg/dl, 30 mg/dl ≤ Lp(a) <50 mg/dl and Lp(a) ≥50 mg/dl had 2.016- and 2.032-fold of CVEs risk than Lp(a) <10 mg/dl in the MUN group. In the MUO group, those with 10 mg/dl ≤ Lp(a) <30 mg/dl, 30 mg/dl ≤ Lp(a) <50 mg/dl, and Lp(a) ≥50 mg/dl all had a significantly higher risk of CVEs than the reference group [NGR plus Lp(a) <10 mg/dl].

**TABLE 2:** Lipoprotein(a) [Lp(a)] and cardiovascular events (CVEs) in different metabolic status.

Metabolic phenotype	HR (95% CI)	
	Unadjusted	Full adjusted
**MHN**		
**Lp(a) per-SD**	1.004 (0.772–1.305)	0.944 (0.717–3.049)
Lp(a) < 10 mg/dL	Ref	Ref
10 mg/dL ≤ Lp(a) < 30 mg/dL	0.618 (0.317–1.208)	0.565 (0.282–1.131)
30 mg/dL ≤ Lp(a) < 50 mg/dL	0.501 (0.188–1.336)	0.553 (0.205–1.490)
Lp(a) ≥ 50 mg/dL	1.021 (0.508–2.052)	0.782 (0.371–1.646)
**MHO**		
**Lp(a) per-SD**	1.244 (0.995–1.557)	1.166 (0.912–1.491)
Lp(a) < 10 mg/dL	Ref	Ref
10 mg/dL ≤ Lp(a) < 30 mg/dL	1.438 (0.667–3.098)	1.245 (0.568–2.729)
30 mg/dL ≤ Lp(a) < 50 mg/dL	*2.393 (1.056–5.424)	*2.438 (1.058–5.626)
Lp(a) ≥ 50 mg/dL	1.842 (0.825–4.112)	1.442 (0.594–3.502)
**MUN**		
**Lp(a) per-SD**	*1.257 (1.090–1.450)	*1.236 (1.063–1.437)
Lp(a) < 10 mg/dL	Ref	Ref
10 mg/dL ≤ Lp(a) < 30 mg/dL	1.514 (0.990–2.316)	1.4880 (0.966–2.292)
30 mg/dL ≤ Lp(a) < 50 mg/dL	*1.895 (1.131–3.177)	*2.016 (1.195–3.399)
Lp(a) ≥ 50 mg/dL	*2.107 (1.309–3.392)	*2.032 (1.236–3.340)
**MUO**		
**Lp(a) per-SD**	*1.307 (1.186–1.441)	1.275 (1.151–1.412)
Lp(a) < 10 mg/dL	Ref	Ref
10 mg/dL ≤ Lp(a) < 30 mg/dL	*1.509 (1.125–2.025)	*1.534 (1.140–2.063)
30 mg/dL ≤ Lp(a) < 50 mg/dL	*1.499 (1.029–2.182)	*1.530 (1.048–2.235)
Lp(a) ≥ 50 mg/dL	*2.399 (1.742–3.303)	*2.206 (1.589–3.062)

*For p < 0.05. Model adjusted for age, sex, body mass index, smoking, family history of coronary artery disease. Gensini score, left ventricular ejection fraction, creatinine, low density lipoprotein cholesterol, and previous use of statin.

## Discussion

The Lp(a)-associated cardiovascular risk in different metabolic phenotypes is not fully determined. In this prospective cohort study on patients with CAD, we found that obese individuals who were metabolically healthy did not have more severe CAD stenosis or higher cardiovascular risk. However, plasma Lp(a) levels were associated with a higher risk of CVEs in both MUO and MUN individuals regardless of obesity. Furthermore, Lp(a) was more strongly associated with CVEs in MUO than in MUN. These findings suggest that Lp(a) may help to stratify cardiovascular risk in patients with metabolic disorders.

In the past half-century, the prevalence of obesity worldwide has almost tripled (2, 13). Obese individuals may suffer from poor quality of life and have reduced life expectancy due to many comorbidities, such as DM, certain types of cancer, and hypertension (14). It is inexcusable that a subgroup of obese people with relatively fewer cardiometabolic risk factors was described as MHO (15). Whether MHO individuals presented a more severe atherosclerosis burden or had a worse prognosis than MHN ones is currently under debate. Previous studies have elucidated this issue in diverse populations. In the study by the International Childhood Vascular Structure Evaluation Consortium, MHO was associated with carotid intima-media thickness (4). Another study by Lin et al. reported that 46.8% of MHO developed into MUO and presented a higher risk of subclinical atherosclerosis (16). The ARIC study concluded that MHO was associated with a worse prognosis but mostly driven by an excess risk of heart failure. In our study population, 5,089 patients with angiography-proven CAD were followed up for 7 years, and we found that MUN and MUO but not the MHO population had a higher risk of CVEs, suggesting that metabolic health status but not obesity influences the risk of CVEs as well as coronary severity.

Large genetic epidemiological studies have documented an increase in the association of high Lp(a) levels and identified corresponding LPA risk genotypes with a higher risk of CAD. In previous studies, a linear relation between Lp(a) and cardiovascular disease was found in those who reached optimal LDL-C levels (17). In some high-risk patients, recurrent ASCVD events occur despite aggressive LDL-C lowering, which may be attributed to Lp(a)-hyperlipoproteinemia. As an inherited risk factor, Lp(a) is especially significant in younger individuals and proved to be predictive of premature MI (18). However, both dietary intervention and environmental changes had a minor effect on plasma Lp(a) levels. Differences in the Lp(a)-associated risk were identified when patients also had other metabolic risk factors. A similar phenomenon was reported in various literature. When participants were categorized by hypertensive or impaired glucose metabolism status, Lp(a) levels were not related to metabolic control but showed increasing associations with CVEs in patients combined with DM or hypertension (19–21). The International Diabetes Federation’s consensus group had given recommendations to better characterize the underlying diseased conditions beyond those superficial factors of metabolic syndrome (22). In this case, Lp(a) may be a crucial marker that not only represents the lipid disorders but also suggests the proinflammatory and prothrombotic state of individuals with obesity and/or metabolic disorder. To the best of our knowledge, we first reported the different associations between Lp(a) and CVEs in four different metabolic phenotypes.

The American Heart Association (AHA) recently issued a statement confirming that Lp(a) is a genetically determined, causal, and prevalent risk factor for CAD (23). Compelling evidence supported the necessity for the screening of cardiovascular risk by Lp(a) measurement (24). The European Society of Cardiology (ESC) and European Atherosclerosis Society (EAS) lipid guidelines also emphasized the importance of Lp(a) measurement in individuals with intermediate risk to further improve the risk stratification (25). In our study population of very high-risk patients with angiography-proven CAD, Lp(a) was reported to play a causal role in the worse prognosis in MUO and MUN individuals. Hence, a reassessment of Lp(a) levels during treatment is greatly necessary for those patients. As the randomized clinical trials to assess the effects of antisense oligonucleotide drugs targeting Lp(a) are in development, this might be a treatment option for very high-risk CAD patients with Lp(a)-hyperlipoproteinemia and metabolic disorders.

The current study had the following limitations. First, the metabolic phenotype is not a permanent state. Transitions from metabolic healthy to unhealthy or obese to non-obese may occur in part of the study population (26). We should also consider that the lack of association between circulating Lp(a) and CVEs in the MHN and MHO groups may be attributed to the lower baseline cardiovascular risk of these individuals. Second, we measured Lp(a) only at baseline, although the parameter was rarely influenced by food and environment, but the follow-up levels of Lp(a) were reported to affect prognosis, which was associated with statin treatment. Third, we did not assess all the metabolic factors such as parameters of insulin resistance due to the features of patients in our study. Hence, more studies are needed to prove our findings.

## Conclusion

In summary, our study first reported that metabolic unhealthy Chinese patients with CAD had higher CVEs regardless of obesity. Furthermore, Lp(a) was a valuable marker for worse prognosis in those who were metabolically unhealthy. Finally, stronger associations were found between Lp(a) and CVEs in those individuals with MUO than in individuals with MUN. Thus, Lp(a) measurement may be clinically valuable for assessing CAD risk in metabolically unhealthy individuals.

## Data Availability Statement

The raw data supporting the conclusions of this article will be made available by the authors, without undue reservation.

## Ethics Statement

The studies involving human participants were reviewed and approved by the Fu Wai Hospital and National Center for Cardiovascular Diseases. The patients/participants provided their written informed consent to participate in this study.

## Author Contributions

J-LJ completed the project, analyzed the data, and wrote the manuscript. J-JL designed the study, interpreted the data, and contributed to critically revising the manuscript. H-WZ contributed to the data collection. N-QW, C-GZ, and Y-LG contributed to the recruitment of patients and the clinical diagnosis of disease and data collection. QD contributed to the collection of clinical data and procedure of laboratory examination. All authors have approved the final manuscript.

## Conflict of Interest

The authors declare that the research was conducted in the absence of any commercial or financial relationships that could be construed as a potential conflict of interest.

## Publisher’s Note

All claims expressed in this article are solely those of the authors and do not necessarily represent those of their affiliated organizations, or those of the publisher, the editors and the reviewers. Any product that may be evaluated in this article, or claim that may be made by its manufacturer, is not guaranteed or endorsed by the publisher.
