# Supplementary material for: Lipoprotein(a) and Cardiovascular Outcomes in Patients With Coronary Artery Disease and Different Metabolic Phenotypes
Source: Front Cardiovasc Med. 2022 May 20;9:870341. doi: 10.3389/fcvm.2022.870341 (PMC9163309; doi:10.3389/fcvm.2022.870341)
Supplement: Supplementary file 1 [file Data_Sheet_1.docx]

**Supplementary Data**

**Supplemental Table S1** Relative cardiovascular risk in different metabolic status

| **Metabolic Phenotype** | **HR (95%CI)** | |
| --- | --- | --- |
|  | **Unadjusted** | **Full Adjusted** |
| **MHN** | Ref | Ref |
| **MHO** | 1.115(0.760-1.634) | 1.163(0.784-1.723) |
| **MUN** | *1.496(1.092-2.050) | *1.414(1.024-1.953) |
| **MUO** | *1.634(1.220-2.190) | *1.747(1.295-2.363) |

* for p<0.05

Model adjusted for age, sex, body mass index, smoking, family history of coronary artery disease. Gensini score, left ventricular ejection fraction, creatinine, low density lipoprotein cholesterol, and previous use of statin.

**Supplementary Data**


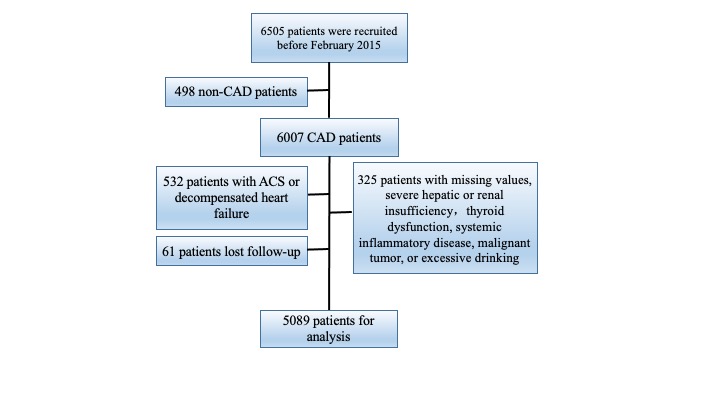
**Supplemental Figure S1** Flowchart of the study
